# Human Embryonic Stem Cell-Derived Progenitors Assist Functional Sensory Axon Regeneration after Dorsal Root Avulsion Injury

**DOI:** 10.1038/srep10666

**Published:** 2015-06-08

**Authors:** Jan Hoeber, Carl Trolle, Niclas Konig, Zhongwei Du, Alessandro Gallo, Emmanuel Hermans, Hakan Aldskogius, Peter Shortland, Su-Chun Zhang, Ronald Deumens, Elena N. Kozlova

**Affiliations:** 1 Uppsala University, Department of Neuroscience, Husargatan 3, 75124 Uppsala, Sweden; 2 Waisman Center, University of Wisconsin, 1500 Highland Avenue, Madison, WI 53705, USA; 3 Institute of Neuroscience, Université Catholique de Louvain, Avenue Hippocrate 54, 1200, Brussels, Belgium; 4 Medical Sciences, School of Science and Health, University of Western Sydney, Campbelltown Campus, Narellan Road, NSW 2567, Australia

## Abstract

Dorsal root avulsion results in permanent impairment of sensory functions due to disconnection between the peripheral and central nervous system. Improved strategies are therefore needed to reconnect injured sensory neurons with their spinal cord targets in order to achieve functional repair after brachial and lumbosacral plexus avulsion injuries. Here, we show that sensory functions can be restored in the adult mouse if avulsed sensory fibers are bridged with the spinal cord by human neural progenitor (hNP) transplants. Responses to peripheral mechanical sensory stimulation were significantly improved in transplanted animals. Transganglionic tracing showed host sensory axons only in the spinal cord dorsal horn of treated animals. Immunohistochemical analysis confirmed that sensory fibers had grown through the bridge and showed robust survival and differentiation of the transplants. Section of the repaired dorsal roots distal to the transplant completely abolished the behavioral improvement. This demonstrates that hNP transplants promote recovery of sensorimotor functions after dorsal root avulsion, and that these effects are mediated by spinal ingrowth of host sensory axons. These results provide a rationale for the development of novel stem cell-based strategies for functionally useful bridging of the peripheral and central nervous system.

Avulsion of spinal roots results in a seriously disabling condition, involving paralysis, loss of sensation and often chronic, intractable neuropathic pain. Partial function of paralyzed proximal muscles can sometimes be achieved by implantation into the spinal cord of the avulsed ventral roots, which offers the injured motor neuron axons access to a peripheral nervous environment^1^. In contrast, to restore sensory functions, avulsed dorsal root axons have to regenerate from a permissive peripheral environment into the hostile, non-permissive spinal cord milieu. Furthermore, dorsal root avulsion (DRA) results in a marked inflammatory response and neuronal loss in the dorsal horn, causing additional obstacles for sensory repair^2^,^3^.

Numerous attempts have been made over the years to overcome the challenge presented by the central nervous system (CNS) to axonal regeneration from dorsal roots into the spinal cord. Thus, transected postganglionic sympathetic axons, ventral root motor axons or peripheral axons of dorsal root ganglion (DRG) cells, all of which are known to have a powerful regeneration capacity, have been anastomosed to the cut proximal portion of a dorsal root^4^,^5^,^6^. These studies showed, that whereas the anastomosed fibers regenerate readily in the peripheral compartment of the dorsal root, they cease to grow as soon as they reach the spinal cord surface.

The first demonstration that fibers were able to grow from the periphery into the adult mammalian spinal cord and make functional connections there, came with a novel experimental approach, transplantation of human fetal sensory neurons to the cavity of extirpated, native, adult rat DRG^7^,^8^,^9^. Subsequent studies demonstrated that sensory axon growth into the spinal cord could be achieved after dorsal root crush by intrathecal delivery of neurotrophin (NT)3, or glial cell line-derived neurotrophic factor (GDNF)^10^,^11^, systemic administration of artemin, a member of the GDNF family^12^,^13^, or adenoviral injection of neurotrophic factors such as nerve growth factor (NGF)^14^,^15^,^16^. More recently, improvements of sensorimotor functions have been shown after implantation of olfactory ensheathing cells to rhizotomized dorsal roots^17^.

These studies have all been carried out after cut (rhizotomy) or crush injuries, rather than after true avulsion (tearing) of dorsal roots. Recently, we developed a clinically more applicable dorsal root avulsion injury model^18^,^19^,^20^. Here, for the first time, we exploit the potential of human stem cell transplants in a dorsal root avulsion model to promote functional restoration of the sensory conduit from the periphery into the host spinal cord. Stem cell-based treatment of dorsal root avulsion injury may serve to: i) provide a growth supportive environment at the dorsal root-spinal cord interface, ii) generate neurons that grow axons into the host spinal cord and form a synaptic relay between terminals of dorsal root axons and dorsal horn neurons, and iii) migrate into the host spinal cord, and replace lost dorsal horn neurons. These options were investigated by transplanting human embryonic stem cell-derived neural progenitors (hNPs) to the site of reattachment between avulsed dorsal roots and the spinal cord. The results show that hNP transplants provide a terrain that enables avulsed sensory axons to enter the spinal cord and that this ingrowth is paralleled by partial sensory recovery.

## Results

### *In vitro* differentiation of human embryonic stem cells into transplantable spinal cord progenitors

**Figure 1:**
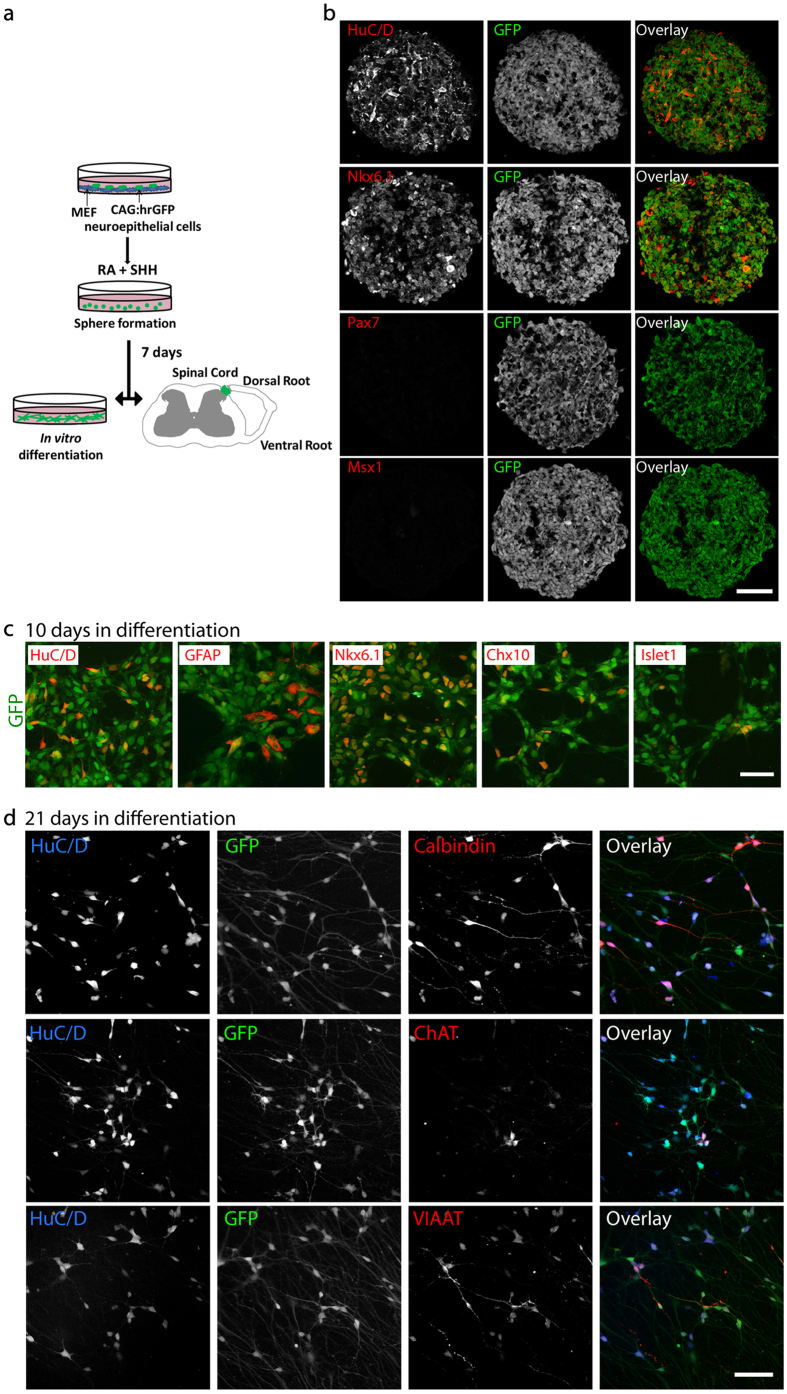
Generation and *in vitro* characterization of transplantable human neural progenitors. (**a**) Outline of the experimental setup. (**b**) At the time point of transplantation, human neural progenitor (hNP) spheres showed expression of HuC/D, a general marker of neurons, and Nkx6.1, a marker of ventral spinal cord progenitors but no expression of the dorsal progenitor markers Pax7 and Msx1. Scale bar, 50μm. (**c**) At 10 days of *in vitro* differentiation, hNP showed expression of HuC/D, the glial cell marker GFAP and several transcription factors indicating a spinal progenitor lineage of neuronal cells (Nkx6.1, Chx10, Islet1). (**d**) At 21 days of *in vitro* differentiation, hNP gave rise to calbindin^+^ interneurons, ChAT^+^ cholinergic neurons and inhibitory neurons indicated by the expression of VIAAT. Scale bar, 20 μm.

Before transplantation, cells were pre-differentiated to spinal cord progenitors. The CMV early enhancer/chicken beta actin promoter – human recombinant green fluorescent protein human embryonic stem cell (CAG-hrGFP hESC) line was generated according to a previously published protocol^21^. In order to direct cells to a spinal progenitor phenotype, we adapted a recently developed protocol for the generation of these cell types within spheres^22^. CAG-hrGFP neuro-epithelial progenitors were dissociated into small clumps and cultured in the presence of 0.1 μM retinoic acid (RA) and 0.2 μM Ag1.3, an agonist of the sonic hedgehog (Shh) pathway. During the first culture day, cells formed spheres, and after seven days these hNP spheres were either directly transplanted into nu/nu mice or used for *in vitro* differentiation assays (Fig. 1a). At this stage, hNPs expressed the neuronal marker HuC/D and the ventral progenitor marker Nkx6.1, but were negative for the dorsal progenitor markers Pax7 and Msx1 (Fig. 1b). For differentiation assays, spheres from day seven were dissociated and plated on laminin.

After ten days, 42.4 ± 2.0% of all cultured cells expressed the neuronal marker HuC/D, 41.3 ± 3.3% expressed the ventral progenitor marker Nkx6.1, 22.8 ± 7.6% expressed the interneuron marker Chx10, 4.7 ± 0.9% expressed the motor neuron progenitor marker Islet1 and 11.97 ± 2.5% expressed glial fibrillary acidic protein (GFAP) (Fig. 1c). After 21 days in culture, different populations of neurons could be identified: 36.8 ± 5.1% of all HuC/D positive neurons expressed the interneuron marker calbindin, 64.9 ± 7.4% expressed vesicular inhibitory amino acid transporter (VIAAT), a marker of inhibitory neurons and 15.8 ± 1.6 expressed choline acetyl transferase (ChAT), a marker of motor neurons and a subpopulation of inhibitory interneurons (Fig. 1d).

## Three months after transplantation

### Characterization of stem cell transplants

The survival and localization of transplanted cells, their differentiation and potential axonal growth into the spinal cord in DRA animals was analyzed three months after transplantation.

**Figure 2:**
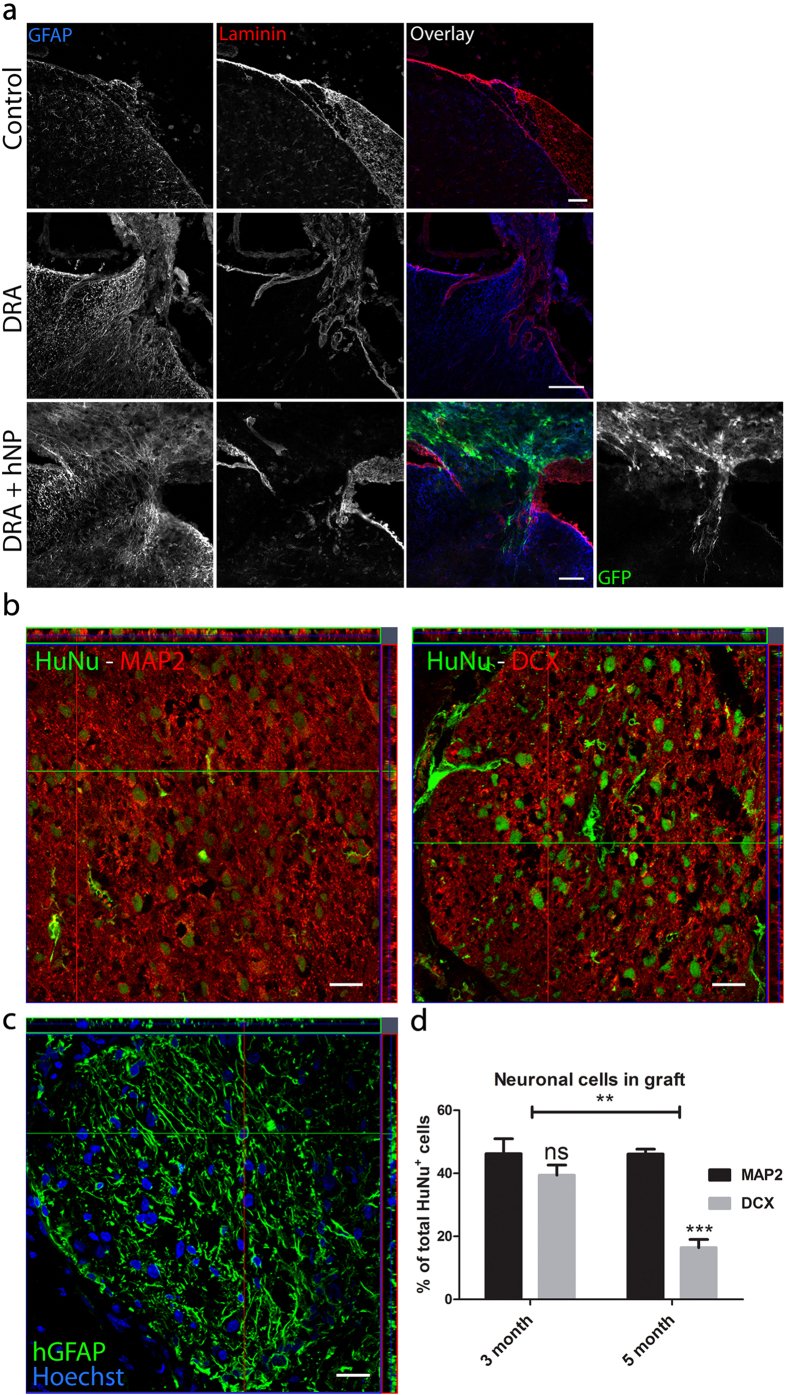
Engrafted hNP differentiated into neurons and glial cells at the site of dorsal root avulsion. (**a**) The dorsal root entry zone was undisturbed by sham operations, whereas dorsal root avulsion (DRA) severely disrupted the spinal cord surface and led to the formation of a glial scar. Engrafted hNP were found in the vicinity of the injured dorsal horn associated with the surface of the spinal cord. Scale bar, 50 μm**. (b+c)** Engrafted hNP at the site of DRA gave rise to neurons characterized by the expression of MAP2 and DCX, and glial cells positive for human-specific GFAP. Scale bars, 25 μm**. (d)** three to five months after transplantation, hNP showed continuous neuronal differentiation indicated by decreasing expression of DCX and stable expression of MAP2. Y-axis shows percent of MAP2^+^ or DCX^+^ cells of the total number of HuNu^+^ cells per section. Data shown in **d** is in mean ± SEM of 3 animals per time point. Asterisks indicate level of statistical significance by two-way ANOVA with Sidak’s multiple comparison (**p < 0.01, ***p < 0.001).

In all animals receiving transplants, GFP-expressing cells were detected on the surface of the spinal cord and in direct vicinity to the areas of DRA, where some invasion into the spinal cord surface could be observed (Fig. 2a). The percentage of detected cells in three months old transplants was around 1.5 ± 0.8% of the total number of transplanted cells. The boundary between the peripheral and central nervous system was visualized by combining stainings for GFAP and laminin. Whereas in the control and avulsed group the GFAP/laminin interface was clearly detected and continuous, in the avulsed group treated with hNP cells this lining was interrupted (Fig. 2a).

Due to variability in the GFP expression of transplanted human stem cells, we used human-specific heat-shock protein 27 (Hsp27) and human nuclear protein (HuNu) as additional markers to label human cells for the analysis of transplanted human progenitor cell differentiation. Virtually all hNPs had differentiated into neuronal or glial phenotypes (Fig. 2b,c). 46.2 ± 3.5% of all cells were microtubule-associated protein 2 (MAP2) positive indicating a neuronal phenotype and 49.8 ± 8.1% expressed human specific GFAP (Fig. 2c). 39.4 ± 3.7% of GFP positive cells expressed doublecortin (DCX) indicating an immature neuronal phenotype (Fig. 2b,d).

**Figure 3:**
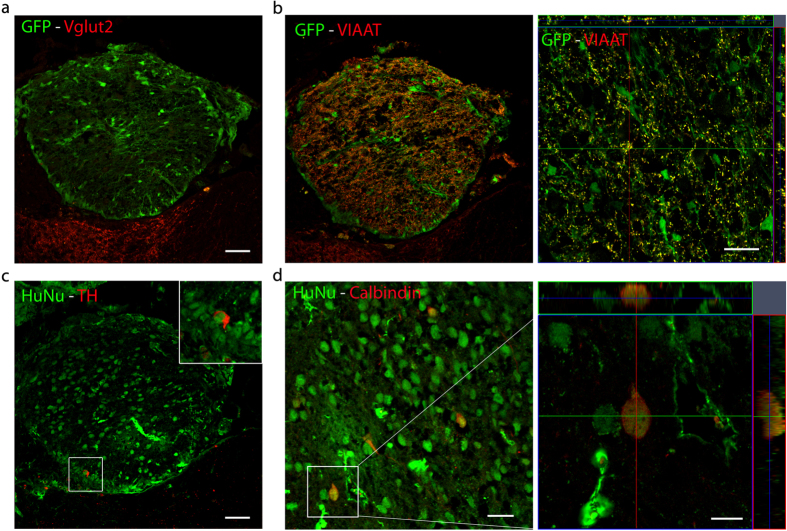
Engrafted human neurons differentiated primarily into inhibitory neurons. (**a**) Engrafted hNP at the site of dorsal root avulsion (DRA) showed no immunoreactivity for Vglut2 . Scale bar, 50 μm. (**b**) Engrafted hNP at the site of DRA showed immunoreactivity for VIAAT localized to GFP^+^ boutons. Scale bars, 50 μm for the transplant overview, 20 μm for the orthogonal projection of a representative area in the transplant. (**c**) hNP at the site of DRA gave rise to a small number of TH^+^/HuNu^+^ cells. Inset shows a high power magnification of the region outlined by the white box. Scale bar, 50 μm. (**d**) Engrafted hNP at the site of DRA gave rise to calbindin^+^/HuNu^+^ interneurons. Scale bar, 25 μm for a representative area in the transplant, 10 μm for the orthogonal projection of the depicted calbindin^+^/HuNu^+^ cell.

No immunoreactivity for vesicular glutamate transporter 2 (VGlut2) was observed in the transplants (Fig. 3a). In contrast, the inhibitory synapse marker VIAAT was expressed in small GFP positive profiles along cells bodies and fibers, indicating that transplanted cells differentiated primarily into inhibitory neurons (Fig. 3b). Underlining this finding, only scattered tyrosine hydroxylase (TH) positive cells could be found (Fig. 3c) whereas hNPs gave rise to interneurons indicated by their expression of calbindin in 5.6 ± 1.1% of all HuNu positive cells in the transplants (Fig. 3d).

### Growth of sensory fibers into the transplant and spinal cord dorsal horn

**Figure 4:**
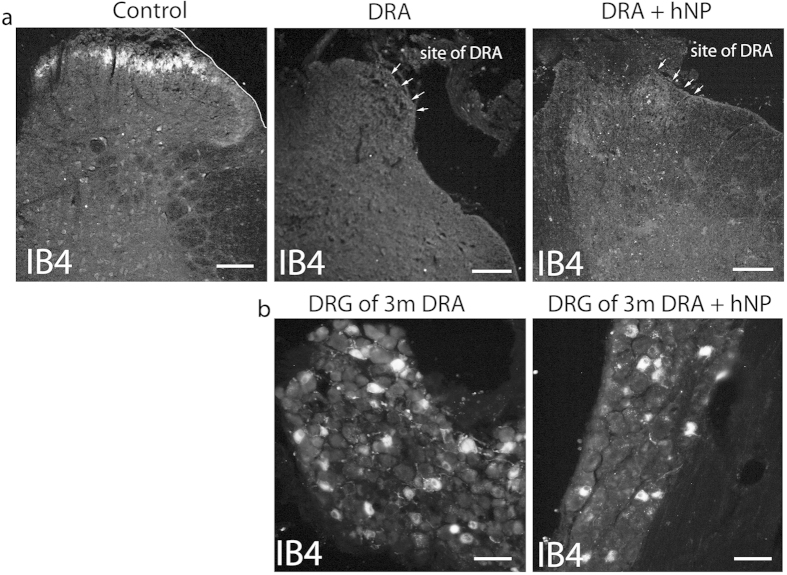
hNP transplantation showed no ingrowth of IB4 labeled sensory fibers in the dorsal horn. **(a)** In control mice, IB4 labels terminals in the superficial dorsal horn. three months after DRA animals that received on transplants, or hNP transplants, showed no transport of IB4 along sensory fibers into the dorsal horn as seen in control animals. (**b**) Retrograde transport of IB4 was confirmed in lumbar DRGs of these DRA and DRA+ hNP transplanted animals. Scale bars, 100 μm for dorsal horn images, 50 μm for DRG image.

**Figure 5:**
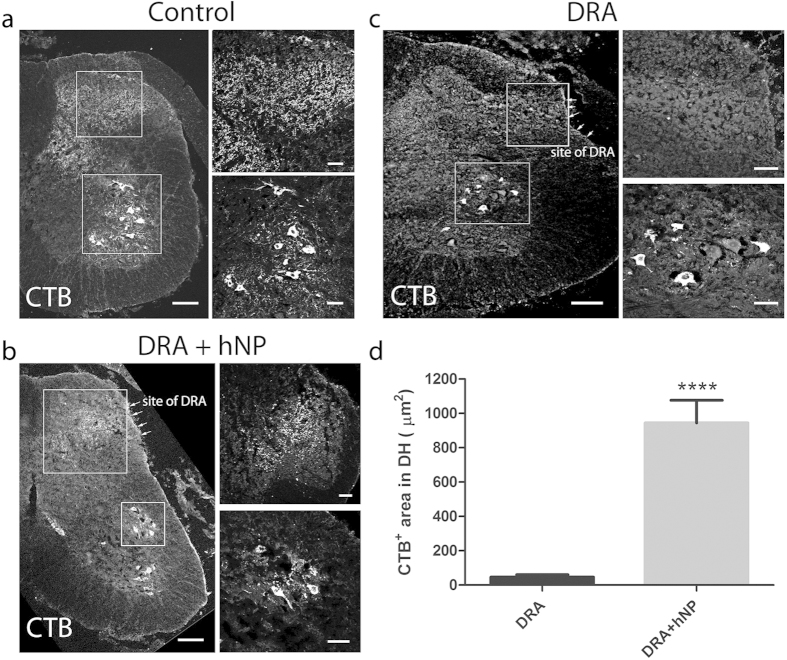
hNP transplantation led to the ingrowth of CTB labeled sensory fibers in the dorsal horn. (**a)** CTB injected into the sciatic nerve of control animals was transganglionically transported to the ipsilateral dorsal horn (DH) and ventral motorneuron pools. (**b**) Animals that received hNP transplanted to the injury site of dorsal root avulsion (DRA) showed CTB labeling in the ipsilateral DH and ventral motorneurons. (**c**) In contrast, animals that underwent dorsal root avulsion (DRA) showed no transport of CTB into the DH but CTB was retrogradely transported to the ventral horn motorneuron pools. Scale bars, 100 μm for juxtaposed images depicting the ipsilateral side of the spinal cord, 50 μm for confocal pictures of ventral and DH areas. (**d**) Immunoreactivity for CTB in the ipsilateral DH along the site of DRA was exclusively found in animals receiving hNP transplants. Y-axis depicts CTB^+^ area in the ipsilateral DH per section. Data shown are in mean ± SEM of 3 animals per condition. Asterisks indicate level of statistical significance by student’s t-test (****p < 0.0001).

Three months after DRA, sensory fibers were traced by injection into the left sciatic nerve with either IB4, which labels a subpopulation of unmyelinated axons^23^, or CTB which labels myelinated axons^24^. Both tracers successfully labeled axons in the appropriate dorsal horn areas in intact animals (Figs. 4a and 5a). However, injections of IB4 did not reveal any labeling through the dorsal root-spinal cord junction site or in the dorsal horn, although numerous neurons were strongly labeled in the lumbar (L)3-5 dorsal root ganglia (DRGs) (Fig. 4a,b).

In contrast, all animals that received an hNP transplant after DRA showed CTB labeled sensory axons projecting into the dorsal horn (Fig. 5b). No CTB labeling was found in the dorsal horn of non-transplanted DRA animals. However, motor neurons ipsilateral in the ventral horn and sensory neurons in the ipsilateral L3-5 DRGs were strongly labeled, indicating that the lack of staining in the dorsal horn was not due to injection failure (Fig. 5c). Quantification of the CTB positive area of the dorsal horn in DRA and DRA+hNP animals revealed a significantly greater CTB immunoreactivity in DRA+hNP animals (n = 3, p < 0.0001, Fig. 5d). Taken together, the tracing studies indicated that myelinated host sensory axons were able to grow into the spinal cord of hNP transplant recipients.

To further examine the distribution of subpopulations of sensory axons at the dorsal root-spinal cord junction, we used antibodies against neurofilament 200kD (NF200) which labels myelinated axons, and calcitonin gene-related peptide (CGRP) which labels peptidergic, non-myelinated axons.

**Figure 6:**
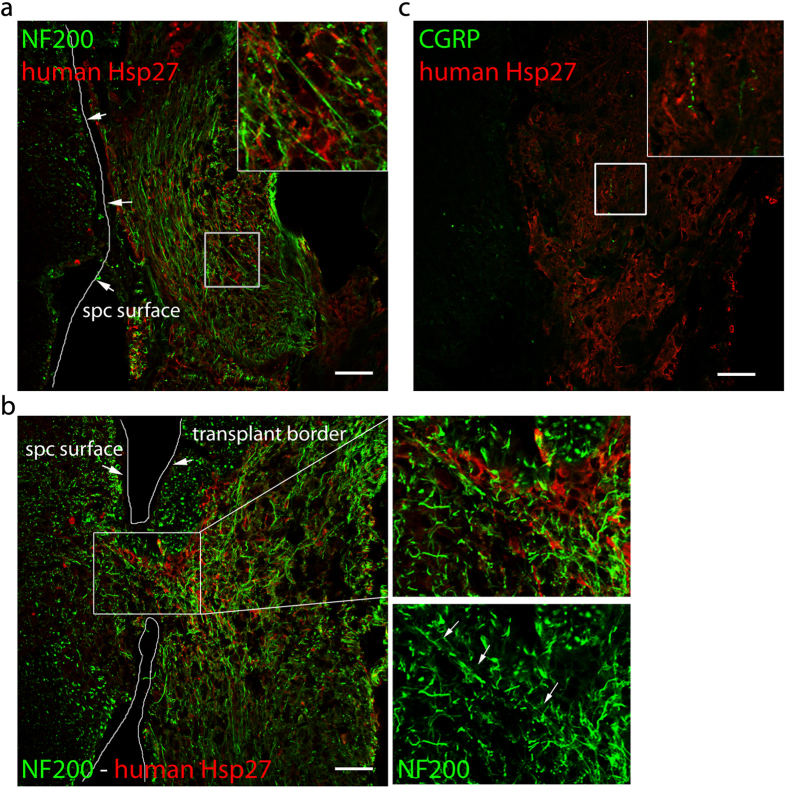
Engrafted hNP provided an environment for the ingrowth of sensory fibers. (**a**) Engraftment sites of hNP showed a large number of parallel NF200^+^ fibers in close proximity to human cells. (**b**) NF200^+^ fibers in close proximity to human cells were able to grow into the dorsal horn. White arrows indicate NF200^+^ fibers passing from the hNP transplant into the spinal cord. (**c**) Only a very small number of CGRP^+^ fibers were found inside the transplant. Cells of human origin were identified by the cytoplasmic distribution of human specific Hsp27. Scale bars; 50 μm. Insets show a high power magnification of the regions outlined by the white box.

In parts of the transplant that did not have access to the spinal cord, we detected extensive ingrowth of NF200 positive fibers into the transplant. These fibers were located next to human Hsp27-expressing fibers that grew in parallel to the spinal cord surface (Fig. 6a).

At the sites of avulsions, NF200 fibers were observed to traverse the spinal cord surface and project into the dorsal horn (Fig. 6b). CGRP labeled axons from the host were also found to grow into the transplant, but were not detected inside the spinal cord (Fig. 6c).

### Behavioral tests of experimental animals

Sensorimotor and somatosensory functions were examined in order to determine whether hNPs transplanted to the site of DRA were able to promote functional recovery, and whether this potential recovery was associated with mechanical hypersensitivity (allodynia), as an indication of abnormal pain processing.

Locomotor performance analyzed using CatWalk analysis was found to be completely unaffected after DRA. Neither interrelated paw parameters (coordination-related) nor individual paw parameters (e.g. base-of-support, paw print intensity, area and the stance duration, swing duration and swing speed) were different between sham-operated, DRA-operated and hNP treated DRA-operated animals (data not shown).

**Figure 7:**
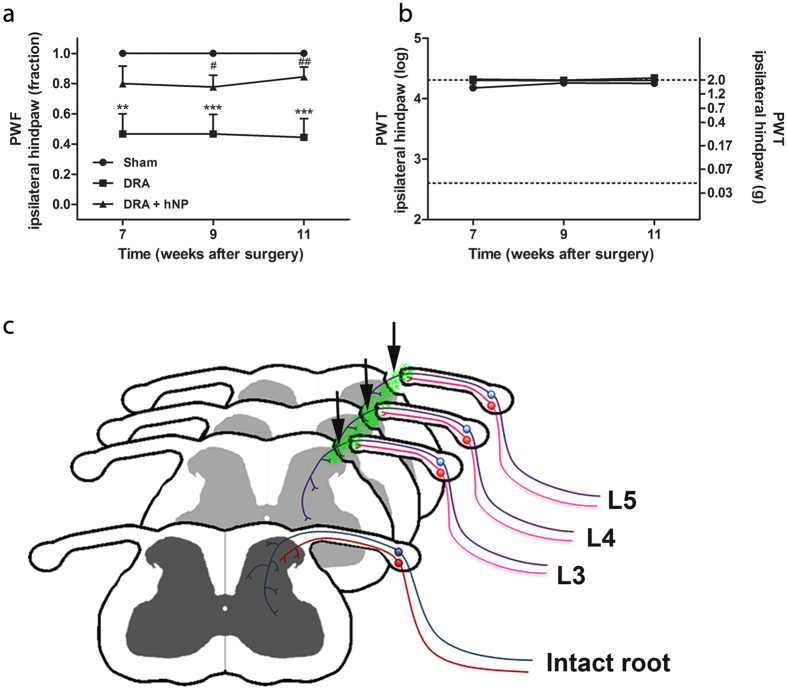
hNP transplantation partially rescued mechanical nociception in dorsal root avulsed mice. (**a**) Dorsal root avulsion (DRA) strongly impaired the mechanical nociceptive function of the ipsilateral hind paw and transplantation of hNP significantly improved this function. (**b**) Neither DRA without treatment nor DRA with hNP transplantation showed mechanical allodynia. * indicates significant difference compared to sham, # indicates significant difference compared to DRA. PWF, paw withdrawal frequency; PWT, paw withdrawal threshold, data shown in a+b are in mean + SEM. Asterisks indicate level of statistical significance by one-way ANOVA with Tukey’s multiple comparison (#,*p < 0.05, ##,**p <0 .01, ***p < 0.001). (**c**) Graphical representation of the hNP graft localization and proposed regeneration of sensory fibers at the site of DRA. In the intact spinal cord myelinated (blue) and non-myelinated (red) sensory fibers project from the dorsal root ganglion via the dorsal root to the spinal cord dorsal horn (frontal image). After hNP (green) transplantation on the top of the avulsed and re-attached L3-L5 dorsal roots (arrows) myelinated fibers traverse the transplants and project to superficial and deep parts of the dorsal horn, whereas non-myelinated fibers grow into the transplant, but fail to enter the spinal cord.

Two mechanosensitivity assays were included in the behavioral monitoring of animals using von Frey hair filaments. First, the paw withdrawal frequency (PWF) to a noxious tactile stimulus was determined as a measure of nociceptive sensitivity (see Material and Methods). This assay revealed a marked reduction in nociceptive sensation in DRA animals one month after surgery. Nociceptive function was tested by applying a 5.5 g von Frey hair filament five consecutive times to the mid-plantar territory of the ipsilateral and contralateral hind paws. While untreated avulsed mice showed a permanent reduction in PWF of 80% as compared to sham-operated mice, this reduction was improved to as little as 20% for mice which received a transplant (Fig. 7a). In the second assay, we investigated mechanical sensitivity using the paw withdrawal threshold (PWT). Untreated as well as treated animals reached the 2.0 g cut-off value or values close to this value, indicating that none of the experimental conditions induced signs of tactile allodynia (Fig. 7b). Moreover, none of the behavioral tests showed deficits for the contralateral hind paw (data not shown). Thus, our analysis of animals surviving three months after transplantation suggested that the improved mechanical nociception in stem cell treated animals was mediated by regeneration of myelinated sensory fibers (Fig. 7c).

## Five months after transplantation

In all animals of the 5 month transplant group GFP-expressing cells were detected on the surface of the spinal cord and in direct vicinity to the areas of DRA similar to the three month group. The percentage of detected cells in 5 month old transplants was 1.2 ± 0.5% of the total number of transplanted cells.

In five months transplant tissue, 46.1 ± 4.0% of all cells expressed MAP2, whereas only 16.4 ± 2.4% expressed DCX. The observed change in DCX expression over time and the stable expression of MAP2 shows the progressive but incomplete maturation of human neurons from three to five months (Fig. 2d). The occurrence of differentiated neuronal types and glia did not change compared to three months transplants (data not shown).

**Figure 8:**
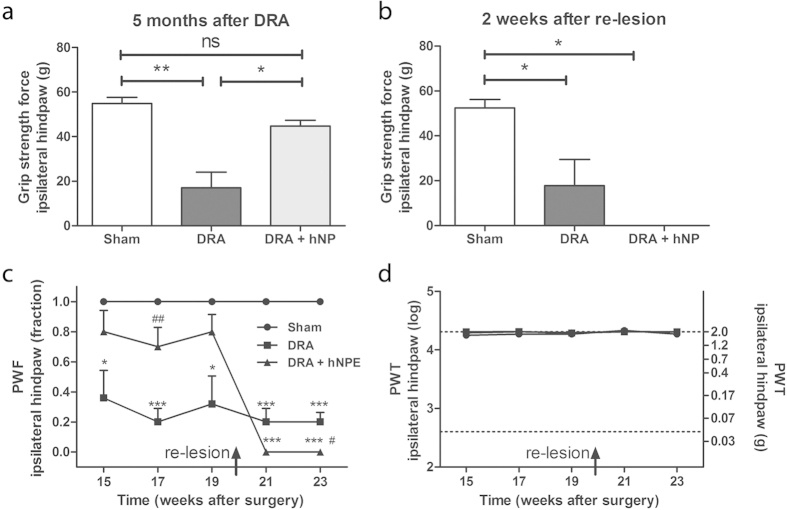
hNP transplantation induced recovery from mechanical nociception was lost after re-lesion of dorsal roots. (**a**) Before re-lesioning of L3-L5 dorsal roots adjacent to transplanted hNP at five months after surgery, transplanted animals showed a statistically significant improvement in ipsilateral hind paw grip strength over non-transplanted (DRA) animals. (**b**) Two weeks after re-lesioning, the ipsilateral grip strength was completely absent. (**c**+**d**) Re-lesoning, completely abolished the restored mechanical nociceptive function and did not lead to mechanical allodynia. * indicates significant difference compared to sham, # indicates significant difference compared to DRA. PWF, paw withdrawal frequency; PWT, paw withdrawal threshold, data shown in a-d is in mean+SEM. Asterisks indicate level of statistical significance by one-way ANOVA with Tukey’s multiple comparison (#,*p < 0.05, ##,**p < 0.01, ***p < 0.001).

To substantiate the interpretation that sensory fibers have regenerated into the spinal cord, long term hNP transplanted animals were tested for nociceptive sensitivity and for sensorimotor function. The functional tests were performed before and after the animals were subjected to section of the L3-5 dorsal roots adjacent to the transplant. For nociceptive sensitivity, the PWF was determined as described for the three months transplant group. For sensorimotor function, animals were subjected to the grip strength test. The latter test measured the tensile force at which the mice released their hind paw’s grip of the apparatus’ grid. Non-transplanted DRA animals demonstrated a strong sensorimotor disturbance and reduced hind paw nociceptive functions (Fig. 8a,c). While sham-operated mice showed release of the hind paw grip at a tensile force of 54.8  ±  2.8 grams, DRA animals did so at 17.1  ±  7.1 grams of tensile force. Transplanted DRA animals showed an intermediate score of 44.8  ±  2.6 grams of tensile force (Fig. 8a). The re-operation to section the L3-5 dorsal roots adjacent to the transplant resulted in a complete loss of grip strength and hind paw nociceptive function two weeks later (Fig. 8b,c). None of the behavioral tests showed deficits for the contralateral hind paw (data not shown). Further, thresholds to mechanical stimulation were still maintained at cut-off values, indicating that mechanical allodynia remained absent (Fig. 8d). These behavioral findings suggested that the partial return of sensorimotor functions in animals receiving hNP transplants was due to regeneration of host sensory axons into the spinal cord which had made connections with the host nociceptive spinal cord circuitry.

## Discussion

In this study, we explored the potential of transplanted human ESC-derived neural progenitors to assist in restoring a sensory conduit from the periphery into the spinal cord in a dorsal root avulsion injury model and to investigate their ability to generate new neurons which may serve as replacements for avulsion induced cell loss in the spinal cord. Our findings support the conclusion that such transplants contribute to partial functional recovery by supporting sensory axon growth into the host spinal cord.

Morphological analysis revealed survival of all transplants during the five months post-operation period, absence of tumor formation and extensive generation of human neurons and glial cells. In all animals, the transplanted cells were located on the surface of the spinal cord with some invasion into the superficial white matter affected by the avulsion injury. However, migration of hNPs into the host spinal cord gray matter was not observed. Thus, transplantation of hNPs at the reattachment site between the gently avulsed dorsal root and spinal cord appear not to provide a source of spinal cord neurons for the replacement of dorsal horn neurons lost due to an avulsion injury.

Transplanted hNPs continued gradual maturation to neural phenotypes between three and five months after transplantation, although even at five months, immature cells still occurred, evidenced by the presence of DCX expressing cells. Interestingly, virtually all neurons in the transplants acquired an inhibitory phenotype and no hNP-derived cells expressing glutamatergic marker Vglut2 were observed. Furthermore, neurons in the transplant did not extend neurites into the host spinal cord gray matter, indicating that they were unable to reach the intrinsic spinal cord circuitries. These findings suggest that the transplant did not act as a relay between host sensory axons and the spinal cord.

At the interface between the transplant and the spinal cord, some areas appeared to be open “gates” between the peripheral and central nervous system, with a reduced concentration of GFAP expressing astrocytes compared to the dense lining of astrocytes at the dorsal root-spinal cord junction after DRA in stem cell untreated animals. These sites were associated with host sensory fibers that traversed the transplant *en route* into the spinal cord. Previously, we showed that embryonic sensory neurons placed into the adult rat DRG cavity extended their fibers into the host spinal cord along blood vessels as bridges to reach the gray matter where they left the blood vessels and extensively arborized and made synaptic connections in the gray matter area^8^. Here, the stem cell transplants also appeared to form bridges, which provided a growth permissive path for regenerating sensory fibers into the host gray matter. This is analogous to the bridges formed by olfactory ensheathing cells that promote sensory regeneration into the spinal cord after dorsal rhizotomy^25^,^26^.

Previous studies have shown that transplanted neural stem cells are able to release a variety of growth promoting molecules, including NGF, brain-derived neurotrophic factor (BDNF), NT3, GDNF and ciliary neurotrophic factor (CNTF)^27^,^28^,^29^,^30^,^31^, which can initiate and support axonal regeneration into the spinal cord. Neural stem cells may also promote angiogenesis, and thereby, indirectly support sensory axon regeneration. The likelihood that one or more of these factors contribute to hNP transplant mediated sensory axon regeneration is in line with previous studies showing that intrathecal or adenoviral delivery of NT3, NGF or GDNF^10^,^11^,^14^,^15^,^16^,^32^, or systemic administration of artemin^13^ immediately after dorsal root crush injury promotes growth of sensory axons into the rat spinal cord. Thus, the combination of reduced glial scar formation at the site of avulsion injury and the potential trophic support from the transplanted cells provided successful ingrowth of host sensory axons into the spinal cord.

CTB tracing and NF200 labeling provided evidence for the regeneration of host sensory axons into the spinal cord. CTB is widely used for tracing myelinated sensory axons^24^. CTB labeled axons extended through the transplant and CTB labeling was present in the dorsal horn of the spinal cord. NF200 is abundantly expressed in myelinated sensory axons^33^,^34^, and NF200 labeled fibers coursed from the host dorsal root through the transplant into the spinal cord. We also labeled non-myelinated peptidergic and non-peptidergic sensory axons with CGRP, and IB4, respectively. Although such axons were present in association with the transplant, we were unable to trace them into the spinal cord. These findings are in line with the previously reported differential growth capacity of myelinated and non-myelinated sensory neurons after a peripheral conditioning lesion. This lesion enhances the growth capacity of sensory neurons with myelinated axons, but not of peptidergic or IB4 binding sensory neurons^35^. The observation that IB4 is transported to the DRG but not into the spinal cord suggests that the lack of labeling is not due to inadequate axonal transport. This observation is consistent with other studies showing a lack of transganglionic transport of IB4 by injured afferents into the spinal cord after nerve injury^23^,^36^. Taken together, these findings indicate that hNP transplants located at the junction between reattached dorsal roots and spinal cord after DRA allow regenerating host myelinated sensory axons to enter the spinal cord and ramify in the dorsal horn gray matter (Fig. 7c). To our knowledge this is the first evidence that hNPs serve as bridges for regeneration of host axons in the adult CNS.

To assess the effect of hNP transplants on sensory functions after DRA, we used a battery of behavioral tests. These tests included the CatWalk gait analysis, which has been reliably and repeatedly used to assess locomotor deficits in animal models of neurotrauma including spinal cord injury and peripheral nerve lesions^37^,^38^,^39^. Surprisingly, DRA did not alter gait for any of the parameters assessed in this assay in neither treated nor untreated animals, suggesting that a functional deficit was not present in ambulating animals. However, non-ambulating DRA animals did show a clear weakness of their ipsilateral hind paw. This deficit could be reliably detected using the grip strength test, which has been previously reported to detect muscle weakness after lesions of forepaw nerves such as the median nerve^40^. In addition to this sensorimotor deficit, DRA resulted in severe loss of nociceptive somatosensory function. The withdrawal frequency to high-intensity mechanical paw stimulation was strongly reduced. The discrete behavioral phenotype induced by DRA shows similarities to what has been described previously after cervical dorsal rhizotomy^41^.

The mouse sciatic nerve is innervated primarily by spinal nerves L3 and L4^42^ with smaller contribution from L5. Avulsion of spinal roots L3-5, confirmed in all animals, should result in a complete loss of all sensations to the hind paw. However, retrograde tracing studies also show a small innervation from adjacent spinal nerves and this probably accounts for the spared sensory innervation^42^. Thus, the behavioral tests assess only relative changes in sensitivity in the tested groups. Indeed, the avulsed group demonstrated reduced nociceptive somatosensory function, which was significantly different to transplanted animals. Likewise, the transplants improved the animal’s ability to sense and react to noxious mechanical pressure. Transplantation of hNPs also resulted in an improvement in grip strength compared to non-transplanted animals as was detected in five months transplants. The read-out for both of these actions is a motor response, implying that sensory information from the hind paw is conveyed to appropriate motor neuron pools for their execution of movement.

The central distribution of CTB labeling in transplant-treated mice is in agreement with the observed behavioral recovery. CTB labeled profiles were detected in deep as well as superficial parts of the spinal cord dorsal horn. CTB labels both high and low-threshold cutaneous mechanoreceptors^43^ as well as proprioceptive axons that terminate throughout the superficial and deep spinal cord laminae, confirming intra-axonal labeling studies^43^,^44^,^45^. Thus, a contribution from CTB labeled regenerating axons to the partial recovery of grip strength and mechanical nociceptive sensation is consistent with this projection pattern and could explain the marked recovery of sensation. Furthermore, relesioning the roots at the transplant interface abolished the behavioral recovery, strengthening these conclusions.

Previous studies have shown that cell transplantation to the injured spinal cord increases the risk for the development of mechanical allodynia, thereby limiting the usefulness of such repair strategies^46^,^47^. Indeed, grafted cells may induce not only plasticity to support functional recovery, but can also support maladaptive plasticity leading to a gain in the nociceptive system^48^. Furthermore, incomplete DRA in combination with stem cell transplants may enhance the risk of such conditions. Mechanical hypersensitivity as measured by changes in the PWT in the von Frey test is widely assumed to reflect mechanical allodynia, a common symptom in neuropathic pain. Since transplanted animals in the present study did not display such hypersensitivity throughout the entire five months postoperative period, we infer that the transplant-mediated repair process does not generate a neuropathic pain-like condition. An important interpretation of this finding is that the hNP transplant approach used here is not associated with creation of pain as a serious side-effect. Taken together, these results show that the transplantation of hNPs to the avulsed and reattached dorsal roots support regeneration of myelinated sensory axons into the host spinal cord and thereby contribute to partial recovery of sensorimotor functions.

This study shows that stem cell transplants improve sensorimotor outcomes following DRA in mice. Although cell transplantation techniques have shown promising results in models of dorsal root injury, to our knowledge, this is the first study using an injury lesion similar to the avulsion injuries encountered in man^18^. The exact mechanism by which transplanted stem cells improve regeneration of avulsed fibers needs further attention, but it is likely that stem cells have several beneficial qualities which may enhance regeneration including modifying the dorsal root-spinal cord interface, inducing growth signals in sensory somata and/or modifying host glial cells from interfering with growth of sensory axons into the adult spinal cord.

## Material and Methods

### Generation and characterization of CAG-hrGFP hESCs

The CAG-hrGFP hESC line was established by the TALEN technology to insert hrGFP (humanized recombinant GFP, Agilent) reporter driven by the CAG promoter (chicken beta-actin promoter with CMV enhancer) in the AAVS1 site^21^. The hESCs were cultured on irradiated mouse embryonic fibroblasts (MEFs) as described in the standard protocol http://www.wicell.org.

### *In vitro* differentiation of hNPs

The CAG-hrGFP hESCs maintained on MEFs were differentiated into primitive neuroepithelial cells under a chemically defined culture system for seven days as described previously^49^. Briefly, after plating overnight, hESCs were switched to neural differentiation medium containing 50% DMEM/F12 (Gibco) and 50% Neurobasal media (Gibco) with 2 mM GlutaMax (Invitrogen) and neuronal supplements 2x B27 (Invitrogen) and 1x N2 (Invitrogen), 3 μM of the GSK3 inhibitor CHIR99021 (Tocris Bioscience) and 2 μM TGFβ/Activin/Nodal receptor inhibitor SB435142 (Tocris Bioscience), 2 μM bone morphogenic protein (BMP), 2 μM ALK2 receptor inhibitor DMH1 (Tocris Bioscience). Further, primitive neuroepithelial cells were patterned into ventral spinal cord progenitors in the presence of 0.05 μM caudalizing morphogen RA (Sigma) and 0.5 μM of the Shh agonist purmorphamine (Phoenix Pharmaceuticals) for seven days. Cells were propagated in the same conditions before pre-differentiation.

Pre-differentiation towards spinal neuronal phenotypes was induced by gently blowing off human cells from the feeder layer, followed by the transfer to a fresh culture dish for sphere formation in the presence of neural differentiation medium supplemented with 0.1 μM RA and 0.2 μM Shh agonist Ag1.3 (Curis). 50% of the medium was changed every second day. After seven days of pre-differentiation, hNP spheres (around one million cells) were placed in polystyrene tubes in culture medium supplied with 10 ng/ml of mimetics of CNTF (Cintrophin) and GDNF (Gliafin)^50^ loaded in nanoparticles to maintain viable conditions for the stem cells before transplantation.

For *in vitro* differentiation, spheres were enzymatically dissociated with the help of TrypLE™ Express (Gibco) and seeded on 0.0001% poly-l-ornithine (Sigma) followed by 20 μg/ml laminin (Sigma) pre-coated coverslips at a density of 1.25 × 10^4^ cells/cm^2^ in ES cell medium supplemented with 1x antibiotic-antimycotic (Invitrogen) and 10 ng/mL of CNTF (Miltenyi Biotec) and GDNF (Miltenyi Biotec). 50% of the medium was changed every other day until cells were fixed in 4% paraformaldehyde in phosphate buffered saline (PBS; 137 mM NaCl, 2.7 mM KCl, 100 mM Na_2_HPO_4_, 18 mM KH_2_PO_4_) after 10 or 21 days of terminal differentiation.

The outcome of terminal differentiation was determined through cellular characterization using immunocytochemistry. Cells on glass coverslips were pre-incubated with blocking solution (1% bovine serum albumin, 0.3% Triton X-100 and 0.1% sodium azide (NaN_3_) in PBS) for 45 mins at room temperature and then incubated overnight at 4 °C with different combinations of primary antibodies: HuC/D, GFAP, Nkx6.1, Chx10, Islet1, GFP, calbindin, ChAT or VIAAT (Table 1).

Cells were washed with PBS, and incubated with the FITC conjugated antibody against green fluorescent protein (GFP-FITC) and corresponding secondary antibodies; Alexa350, Alexa488, Alexa555, Cy3 (Table1) in blocking solution for 1 h at room temperature. After removing the secondary antibody followed by one wash with PBS, cells were stained with Hoechst 33342 in PBS for 5 minutes, washed twice with PBS, once in distilled water and embedded in 8 μl of mounting medium (50% glycerol in PBS and 100 mM propyl-gallate (Sigma) on a glass slide.

### Surgery

All animal experiments were approved by the Uppsala Regional Ethical Committee for Animal Experiments, as required by Swedish Legislation and in accordance with European Union Directives.

Surgery was performed on adult male nu/nu NMRI mice (n = 36; Mollegaard, Denmark) according to the previously described procedure^18^. All animals were 6 weeks old at the start of the experiment and and 8 weeks old animals were subjected to the behavioural tests during the subsequent five months before being sacrificed. Briefly, after an intraperitoneal injection of ketamine (100 mg/kg, Intervet), xylazine (20 mg/kg, Bayer Healthcare) and acepromazine (3 mg/kg, Pharmaxim)^51^, the skin of the lower back was incised in the midline. The paravertebral muscles and fascia were dissected to expose the vertebrae corresponding to the left L3-L5 dorsal root entry points. The vertebral laminae were removed and the dura opened. Topical analgesia (xylocaine) was applied on the spinal cord surface over the L3-L5 dorsal roots, which were subsequently gently pulled from the spinal cord with a pair of forceps so that the rootlets ruptured at the root entry zone. The hNPs were accurately arranged with fine jeweler’s forceps along the avulsed areas of L3-L5 spinal cord. Around 500 000 cells were placed along the spinal cord with access to the avulsed areas. The roots were placed back into the original position on the top of stem cell transplants, making the spheres attached to the spinal cord at these sites. The rest of the spheres located between these places attached poorly and presumably vanished during the early post-transplantation period. Muscles and skin were sutured separately.

For non-transplanted animals, roots were directly re-positioned after the avulsion performed in similar way. In sham-operated animals, the dura and the spinal roots were left untouched after the laminectomy. The muscles and the skin were then sutured in layers using single silk (4.0 and 6.0, respectively) sutures. Animals were allowed to recover in a heated cage and were given 3μg buprenorphine (Temgesic® RB Pharmaceuticals) subcutaneously twice daily for three days.

### Immunohistochemistry

After post-operative survival times of three and five months, mice were terminally anaesthetized and perfused with room temperature saline, followed by a fixative solution containing cooled 4% w/v formaldehyde and 14% v/v saturated picric acid in PBS (pH 7.35 – 7.45). The left L3–L5 spinal cord segments with attached dorsal roots as well as DRGs were removed, post-fixed at 4 °C for 4 hours and then cryo-protected overnight in PBS containing 15% sucrose. Serial coronal sections (14 μm) of the spinal cord, of DRGs and dorsal roots were cut on a cryostat, placed on SuperFrost® Plus glass slides (Menzel-Gläser, Braunschweig, Germany) and processed for immunohistochemistry (see above) with mixed primary antibodies for laminin, GFAP, HuNu, MAP2, DCX, human-specific GFAP (STEM123), VGlut2, VIAAT, TH, calbindin , Hsp27, NF200 or CGRP. The anti-cholera toxin subunit B (CTB, List biological laboratories) antibody was incubated for 4 h at room temperature and the anti-*Griffonia Simplicifolia Agglutinin* isolectin B4 (IB4, Vector Laboratories) antibody was incubated overnight at 4 °C (Table 1). A minimum of three animals for each experimental group were analyzed at the two time points. hNP pheres were collected and fixed with 4% paraformaldehyde in phosphate buffered saline followed by processing for cryo-sectioning (8 μm sections) and immunohistochemistry as stated above with mixed antibodies for HuC/D, Nkx6.1, Pax7 or Msx1.

### Tracing of sensory axons

Transganglionic tract tracing methods were used in animals surviving three months after operation to label and chart the course and projections of sensory fibers in the avulsed dorsal root and corresponding spinal cord segments. We used CTB (List Biological Laboratories) that labels myelinated sensory afferents^24^ and lectin *Griffonia Simplicifolia Agglutinin* isolectin B4 (IB4, Life Technologies, I21413) that labels unmyelinated fibers^23^. For tracer injections, animals were anesthetized with isoflurane® (Baxter) and given a 3 μg dose of buprenorphine subcutaneously at the onset of anesthesia. After a mid-thigh incision, the left sciatic nerve was exposed using blunt dissection after which approximately 50 μl of topical xylocaine (10 mg/ml) was applied on the nerve and surrounding tissue. The nerve was immobilized using proximal and distal silk threads. The CTB or IB4 was diluted to a final concentration of 1% as previously described^52^ and 3 μl were injected via a small hole in the epineurium into the sciatic nerve via a small glass needle, and the wound was closed using single silk 6.0 sutures. The animals were given daily subcutaneous buprenorphine injections post-operatively. After three days, the animals were sacrificed and cryo-sections prepared as described above. Tracers were injected in separate animals (three in each experimental group).

### Microscopy and cell counting

Images of cells on coverslips were captured with a Plan-Apochromat 20x objective (NA 0.75) and images for CTB quantification were taken using a Plan-Apochromat 10 x objective (NA 0.45) attached to a Nikon Eclipse E800 epifluorescence microscope equipped with a Nikon DXM1200F CCD camera. Overlays of multichannel pictures were prepared with Fiji ImageJ2.0.0-rc-2^53^. Cells on coverslips were manually counted with the help of the cell-counter plug in for Fiji ImageJ2. Proportions of specific cell types for the total number of cells after 10 days of terminal differentiation and proportions of post-mitotic neuronal subtypes for the total number of neurons after 21 days of terminal differentiation were calculated for a minimum of 12 images from 3 independent experiments.

Immuno-labeled sections were analyzed using a Zeiss LSM710 confocal laser scanning microscope. Single images were captured using a LD LCI Plan-Apochromat 25x objective (NA 0.8), for orthogonal projections of human GFAP, MAP2 or DCX labeled human cells, images were taken with a Plan-Apochromat 40x objective (NA 1.3) and for orthogonal projections of areas inside the region of the transplant a Plan-Apochromat 63x objective (NA 1.4) was used. Z-stacks were taken with an optical slice thickness of 1 μm at an interval of 1 μm.

### Image processing and statistical analysis

The proportion of MAP2/HuNu/Hoechst or DCX/HuNu/Hoechst triple labeled human cells to the total number of HuNu/Hoechst positive cells per slide was used to analyze the differentiation of transplanted hNPs to neurons and to estimate their degree of maturity over time. The cells were counted using z-stacks covering a spinal cord segment of 14 μm following semi-stereological principles in three and five months old transplants from every 10^th^ slide of 3 animals, respectively. Differences in the MAP2/DCX distribution between the two time points were analyzed using a two-way ANOVA with Sidak’s multiple comparisons test, p < 0.05. The proportion of anti-human GFAP labeled cells of all human cells was quantified by counting all hGFAP/Hoechst cells and HuNu/Hoechst cells in adjacent slices but otherwise identical to MAP2 and DCX quantifications.

The area of CTB immunoreactivity in the dorsal horn of every 10^th^ slide of 3 animals for DRA and DRA+hNP conditions from animals three months after surgery was analyzed. Images for CTB quantification were taken under identical image acquisition settings for both conditions using a Plan-Apochromat 10 x objective (NA 0.45) attached to the epifluorescence microscope setup. For quantification, images were preprocessed in Fiji Image2 by subtracting background using the 488 nm channel and the implemented rolling ball algorithm, followed by setting a manual threshold for CTB positive staining. The dorsal horn was outlined in the raw image and transferred onto the threshold set image, the total area of CTB immunoreactivity in the ipsilateral dorsal horn was measured and differences in the total area of immunoreactivity were analyzed using a t-test, p < 0.05. No quantification of IB4 tracer injection was performed as no labeling in the spinal cord of both avulsion groups (treated and untreated with stem cells) was found.

We roughly estimated the rate of cell survival in the transplants after three and five months. The amount of HuNu positive nuclei was counted on every 10^th^ section through the transplants using z-stacks covering a spinal cord segment of 14 μm following semi-stereological principles. The mean number was calculated for each animal and then for all experimental groups with three and five months survival after transplantation.

All statistical analyzes were performed in GraphPad Prism 5.04.

### Behavioral analyzes

Behavioral testing was done to assess locomotor performance and sensorimotor function (CatWalk gait analysis at early post-transplantation stage and grip strength test at late post-transplantation stage) and nociceptive sensory function (determination of paw withdrawal frequency, PWF). In addition, the putative development of mechanical allodynia as a sign of abnormal pain processing was determined with the paw withdrawal threshold, PWT. Behavioral assessments were performed in three groups of animals: sham-operated (n = 9), DRA-operated (n = 8), and DRA-operated with transplants (n = 9).

CatWalk gait analysis was selected for its ability to detect locomotor deficits in a range of neurotrauma models^37^,^38^,^39^. The system, which was placed in a darkened room, consists of a 1.5 m-long runway in which the glass floor is internally illuminated. Upon paw contact with the glass floor, the paw print becomes illuminated and is detected by a high speed color video camera positioned underneath the runway. Each mouse was separately positioned at one end of the runway and once an uninterrupted run (without hesitation or intermediate stop) was made from one end of the runway to the other, the run was recorded and the resulting file saved on a computer equipped with analysis software (CatWalk software 7.1). After each mouse had completed three such runs with minimally four step cycles in every run (i.e. each paw was placed minimally four times), analysis of six parameters (base-of-support, paw print intensity, paw print area, stride length, stance phase and swing phase), typically used for describing gait abnormalities after neurotrauma in rodents were performed. Data was expressed as a percentage of the values of sham-operated mice.

Two mechanosensitivity assays were included in the behavioral monitoring of this investigation. These assays both relied on von Frey hair filaments. In the first assay, PWF to a suprathreshold mechanical stimulus was determined. The PWF was used as a measure of nociceptive sensory function using a 5.5 g-filament. This filament was applied five consecutive times to the glabrous mid-plantar hind paw surface with an inter-stimulus interval of at least 5 seconds. The ratio of positive paw withdrawal out of the five filament applications was determined. In the second assay, a set of seven hair filaments was used (0.04, 0.07, 0.16, 0.4, 0.6, 1.0, and 2.0 grams) to determine the PWT using the up-down method^54^, a sensitive method to assess putative mechanical allodynia after nerve injury. Herein, the glabrous mid-plantar hind paw surface was stimulated starting with the 0.4 g-filament; a positive withdrawal was followed by a stimulation with the lower-next filament, while a negative response was followed by a stimulation with the higher-next filament. This procedure was continued until five stimulations were delivered after the first positive paw withdrawal response. On the basis of the data, the 50% PWT was calculated. The PWT was used as a measure of mechanosensitivity in order to detect a putative hypersensitivity to mechanical stimuli in the postoperative period.

Non-ambulatory sensorimotor function was assessed using the grip strength test. In this test, each mouse was gently held upright by the scruff of the neck and then positioned above the lateral aspect of the grid facing towards the meter of the apparatus until the animal gripped the grid with either one of the hind paws. The animal was then gently pulled back horizontally to the meter and the tension force at which the grip was released by the animal was measured. Measurements were repeated two more times and the average grip strength (in grams) was calculated for each hind paw of each animal at each time point of investigation. In order to reduce stress of the animal during the procedure, the animal was habituated to the procedure before taking the actual measurements. Data was expressed in the gram-force required for the release of the hind paw from the grid.

After the behavioral assessment at five months, DRA animals with transplants underwent a second surgery at which L3-L5 dorsal roots were cut distal to the transplant. Two weeks later, the animals were re-assessed in the behavioral assays.

## Additional Information

**How to cite this article**: Hoeber, J. *et al*. Human Embryonic Stem Cell-Derived Progenitors Assist Functional Sensory Axon Regeneration after Dorsal Root Avulsion Injury. *Sci. Rep*. **5**, 10666; doi: 10.1038/srep10666 (2015).

## Figures and Tables

**Table 1:** Antibodies used for immunohistochemistry.

Antigen	Host	Cat no.	Source	Dilution
***Primary***				
Calbindin	Rabbit	CB-38a	Swant	1:2000
CGRP	Goat	ab36001	Abcam	1:200
ChAT	Goat	AB144P	Millipore	1:100
Chx10	Sheep	AB9016	Millipore	1:400
CTB	Goat	703	List	1:100
DCX	Rabbit	ab18723	Abcam	1:1000
GFAP	Rabbit	2016-04	DAKO	1:500
GFAP	Mouse	MAB3402	Millipore	1:200
GFP	Mouse	A11120	Molecular Probes	1:100
Hsp27	Rabbit	SPA-803	Nordic Bioscience	1:300
HuC/D	Mouse	A21271	Novex (Life Technologies)	1:400
HuNu	Mouse	MAB1281	Chemicon	1:50
IB4	Goat	AS-2104	List	1:100
Islet1	Mouse	40.2D6	DSHB	1:200
Laminin	Rabbit	L9393	Sigma	1:50
Map2	Chicken	ab5392	Abcam	1:1500
Msx1 + 2	Mouse	AB 531788	DSHB	1:50
Nkx6.1	Mouse	F55A10	DSHB	1:20
Pax7	Mouse	PAX7	DSHB	1:50
RT97 (NF200)	Mouse	1178709	Mannheim Boehringer	1:50
Stem123 (huGFAP)	Mouse	AB-123-U-050	StemCells	1:1000
TH	Rabbit	P40101	Pel-Freez	1:100
VGluT2	Guinea Pig	AB2251	Millipore	1:5000
VIAAT	Rabbit	131 002	Synaptic Systems	1:100
***Secondary***				
Alexa 350	Mouse	A10035	Invitrogen	1:500
Alexa 488	Mouse	A21202	Invitrogen	1:1000
Alexa 488	Rabbit	A11008	Invitrogen	1:1000
Alexa 546	Chicken	A11040	Invitrogen	1:1000
Alexa 555	Goat	A21432	Invitrogen	1:1000
Alexa 555	Rabbit	A31572	Invitrogen	1:1000
Alexa 647	Mouse	A31571	Invitrogen	1:1000
Cy3	Guinea Pig	706-165-148	Jackson	1:1000
Cy3	Mouse	715-165-151	Jackson	1:1000
